# Non-communicable diseases research output in the Eastern Mediterranean region: an overview of systematic reviews

**DOI:** 10.1186/s12874-020-00924-0

**Published:** 2020-03-20

**Authors:** Alaa Akkawi, Joanne Khabsa, Aya Noubani, Sarah Jamali, Abla M. Sibai, Tamara Lotfi

**Affiliations:** 1 Department of Epidemiology and Population Health, Faculty of Health Sciences, American University of Beirut, Beirut, Lebanon; 2 Clinical Research Institute, American University of Beirut Medical Center, Beirut, Lebanon; 3 Global Health Institute, American University of Beirut, Beirut, Lebanon; 4 Faculty of Medicine, American University of Beirut, Beirut, Lebanon; 5 Department of Internal Medicine, Faculty of Medicine, American University of Beirut, Beirut, Lebanon

**Keywords:** Research methodology, Review of reviews, Overview, Systematic reviews, Meta-analysis, Non-communicable diseases, Research agenda, Eastern Mediterranean region

## Abstract

**Background:**

Rates of non-communicable diseases (NCDs) are rapidly rising in the Eastern Mediterranean Region (EMR). Systematic reviews satisfy the demand from practitioners and policy makers for prompt comprehensive evidence. The aim of this study is to review trends in NCD systematic reviews research output and quality by time and place, describe design and focus, and examine gaps in knowledge produced.

**Methods:**

Using the Montori et al. systematic reviews filter, MeSH and keywords were applied to search Medline Ovid, Cochrane Central and Epistemonikos for publications from 1996 until 2015 in the 22 countries of the EMR. The ‘Measurement Tool to Assess Systematic Reviews’, AMSTAR, was used to assess the methodological quality of the papers.

**Results:**

Our search yielded 2439 papers for abstract and title screening, and 89 papers for full text screening. A total of 39 (43.8%) studies included meta-analysis. Most of the papers were judged as being of low AMSTAR quality (83.2%), and only one paper was judged as being of high AMSTAR quality. Whilst annual number of papers increased over the years, the growth was mainly attributed to an increase in low-quality publications approaching in 2015 over four times the number of medium-quality publications. Reviews were significantly more likely to be characterized by higher AMSTAR scores (±SD) when meta-analysis was performed compared to when meta-analysis was not performed (3.4 ± 1.5 vs 2.6 ± 2.0; *p*-value = 0.034); and when critical appraisal of the included studies was conducted (4.3 ± 2.3 vs 2.5 ± 1.5; p-value = 0.004). Most of the reviews focused on cancer and diabetes as an outcome (25.8% and 24.7%, respectively), and on smoking, dietary habits and physical activity as exposures (15.7%, 12.4%, 9.0%, respectively). There was a blatant deficit in reviews examining associations between behaviors and physiologic factors, notably metabolic conditions.

**Conclusions:**

Systematic reviews research in the EMR region are overwhelmingly of low quality, with gaps in the literature for studies on cardiovascular disease and on associations between behavioral factors and intermediary physiologic parameters. This study raises awareness of the need for high-quality evidence guided by locally driven research agenda responsive to emerging needs in countries of the EMR.

## Background

Non-communicable diseases (NCDs) are the leading causes of morbidity and mortality worldwide, with disproportionately higher rates in low- and middle-income countries (LMIC) [1]. In 2010, NCDs were responsible for 38 million (68%) of the world’s 56 million deaths and for the majority of premature deaths (82%) [2]. The WHO projects that NCD deaths will increase globally by 17% in the next 10 years, with the second greatest increase seen in the Eastern Mediterranean region (EMR) (25–27%) [3]. In the EMR, NCDs feature prominently in the top ten causes of death, and the age-standardized death-rate in most countries is now over 300 per 100,000, placing a huge burden on already overstretched health systems [4].

Accordingly, the past two decades have seen a growing international recognition of the importance of developing national policy frameworks for NCD prevention and control [5]. In 2010, the WHO published its “Package of Essential NCD Interventions for Primary Healthcare in Low-resource Settings” [6]. One year later, it issued two reports that outlined country capacities to respond to the NCD epidemic [7, 8]. The high-level UN meeting in 2011 described NCD as a developmental issue and declared the need to set up a road-map for NCD surveillance, prevention and management, focusing on best available evidence [2]. More recently, attention has been drawn to the importance of high-quality research in the EMR, including implementation research and evaluation studies for evidence-based policy-making [4].

Systematic reviews have the potential to provide high-quality evidence to support informed interventions. Yet, these types of studies are largely lacking from LMIC and in the EMR [9], and one cannot simply assume that the evidence coming from one setting can be extrapolated to another. When selecting an intervention program, consideration should be given to several context-specific variables such as feasibility, implementation capacity, effectiveness, and affordability according to national conditions and resources [10]. Whilst for some questions, evidence from other settings would be as important to include to inform decision-making, locally driven practice-based health research and relevant high-quality evidence remain critical for assessing barriers to and enablers for implementing internationally recommended strategies and examining the integration of best practices into local healthcare settings [11].

This study was set to answer the question: what is the extent and quality of existing evidence based on systematic reviews examining NCD in the EMR? Reviews of reviews aim at filtering the information overload, improve access to targeted information and support decision-making [12]. These have scarcely begun to be explored in the global literature, with one study relying solely on the Cochrane database of systematic reviews [13] and another covering a single NCD condition, diabetes [14]. Previous NCD reviews in the region have either had broader scope of inclusion criteria incorporating studies of various methodologies including primary studies [9], or were focused on a distinct theme in NCD research (e.g cost analyses or genetics in NCD research) [15, 16] or a single country [17]. However, no previous study has appraised NCD-related evidence generated from exclusively systematic reviews. In this study, we conduct a systematic review of the NCD research landscape of systematic reviews, with a lens of examining gaps and strengths in the knowledge produced. Guided by overview of systematic reviews framework [18, 19], we map the NCD research productivity of systematic reviews published in the past 20 years in all countries of the EMR, appraise their quality by time and place, describe design, setting and focus, and identify evidence gaps in the published literature.

## Methods

### Study protocol

The protocol for this study was specified in advance and registered in PROSPERO (CRD 42017054145) (http://www.crd.york.ac.uk/PROSPERO/display_record.asp?ID=CRD42017054145). We followed the PRISMA reporting guideline.

### Data sources and search strategy

Owing to the scarcity of systematic reviews prior to 1990, we limited our search to the reviews published in the past 20 years (1996–2015). We conducted an electronic search of Medline in April 2016, Cochrane Central in May 2016 and Epistemonikos in May 2016. We searched for systematic reviews using a systematic review filter provided by Montori and colleagues [20]. For the definition of NCDs and NCD risk factors, we used the WHO Global Action Plan for the Prevention and Control of NCDs 2013–2020 framework [21]. This definition classifies NCDs into four major conditions: cardiovascular diseases (CVDs), cancer, diabetes mellitus (DM), and chronic respiratory diseases (CRDs), within a framework of a casual pathway of primarily four main risk behaviours (tobacco use, unhealthy diet, physical inactivity, and the harmful use of alcohol) mediated by four metabolic/physiological changes (raised blood pressure, overweight/obesity, raised blood glucose and raised cholesterol). A comprehensive search was developed in consultation with an experienced librarian using the following as both indexed MeSH terms and free text words: neoplasm, chronic obstructive pulmonary disease, asthma, pulmonary emphysema, cardiovascular disease, cardiovascular abnormalities, cardiovascular infections, cardiovascular pregnancy complications, vascular disease, heart diseases, glucose metabolism disorders, diabetes mellitus, lipid metabolism syndrome, metabolic syndrome and its associated components, body weight changes, tobacco use, food habits, physical activity and alcohol. We used the population of the countries served by the WHO Regional Office for the Eastern Mediterranean to define the EMR. This comprises the 22 Member States (Afghanistan, Bahrain, Djibouti, Egypt, Iran, Iraq, Jordan, Kuwait, Lebanon, Libya, Morocco, Occupied Palestinian territory, Oman, Pakistan, Qatar, Saudi Arabia, Somalia, Sudan, Syria, Tunisia, United Arab Emirates and Yemen), with a population of nearly 580 million people [22]. Articles were included regardless of the domain of the research question (therapeutic, diagnostic or etiology). In this paper and in line with the most commonly used terminology, we use hereafter the term ‘overview’ to describe our study employing the ‘systematic review of systematic reviews’ methodology and framework [12]. The detailed search strategies for the three search engines are provided in Supplementary file 1.

### Eligibility criteria

We included in this study articles that explicitly stated the use of the ‘systematic review with/without meta-analysis’ methodology in the title/abstract and explicitly reported the use of at least one database when conducting the search. Studies were excluded when full text was not available; if published in languages other than English or French; when findings were aggregated and hence could not be tagged to individual studies or individual countries; when the population of interest were emigrants living outside EMR countries; and if less than 30% of the primary studies included in the review targeted EMR countries. The choice of the 30% was made in consultation with experts in the field and was found to provide the best balance for sensitivity and specificity for inclusion and exclusion of studies, while maintaining a good representation of papers with EMR relevant data.

### Study selection and data extraction

Following a calibration exercise to ensure definitions are clear to all team members, three teams of two reviewers each screened, in duplicate and independently, firstly the titles and abstracts of the identified citations, and secondly the full text, based on the eligibility criteria. Discordances were resolved by discussion among the team members; and in case of disagreement, a third reviewer was consulted. A data abstraction form was developed, piloted and subjected to two rounds of a calibration exercise to ensure reliability of the tool and harmonization of the abstraction process among reviewers. Using Excel sheets, the following data were extracted, in duplicate, from each full text study review:

- Year of publication
- First and corresponding authors’ institutional affiliation and country. In this study, we focused on corresponding author affiliation
- Setting, that is country/countries of study population, detailing the various countries for each primary study included in the review
- Study design (systematic review with/without meta-analysis)
- Total number of studies included in the review
- Type of the primary studies included in the review (observational/ intervention/others including genetic and qualitative studies)
- NCD themes and topics appraised including CVDs, DM, cancer, CRDs, obesity, hypertension, hyperlipidemia, metabolic syndrome (METs), smoking, diet, physical activity, and alcohol), and whether these were considered in the publication as intervention/exposure or the outcome variables
- Critical appraisal of included studies
- Heterogeneity assessment and publication bias were examined on their own, but reported as part of overall assessment of the quality of the paper as presented below, and finally
- Funding source

In addition to the above, the quality of the systematic review paper was assessed using the “Measurement Tool to Assess Systematic Reviews (AMSTAR)” tool [23]. AMSTAR includes a list of 11 items and was judged to have good face and content validity for measuring the methodological quality of systematic reviews [23]. A score was allocated for each item (1 for ‘yes’ and 0 for ‘no’), with a higher score indicating better quality. Articles were categorized based on the summative AMSTAR score as low (0–4), medium (5–8) or high-quality (9–11) [24]. Queries during data extraction process were resolved by a third researcher, and responses were shared with all teams undertaking data abstraction.

### Data analyses

Data sheets were then exported into SPSS for analyses. Descriptive statistics were conducted to determine the distribution of the included studies by quality, exposure/intervention and outcomes, and consequently the evidence gap map.

## Results

**Fig. 1:**
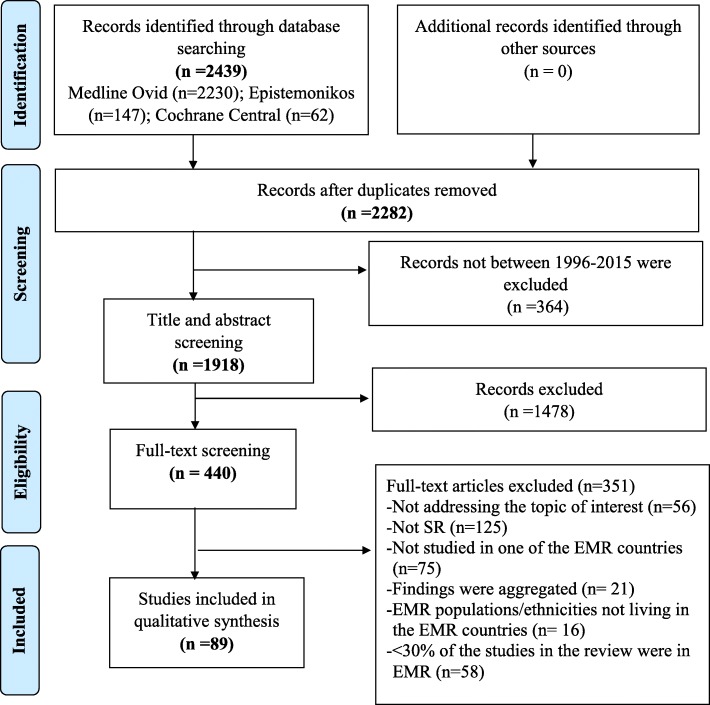
PRISMA study flow chart

Our search initially retrieved a total of 2439 records, the majority of which appeared in Medline Ovid (2230 records), followed by Epistemonikos (*n* = 147) and Cochrane Central (*n* = 62). After exclusion of duplicates and those published outside the scope of the study period, a total of 1918 unique and potentially relevant systematic review publications were retained for screening. We excluded 1478 based on title and abstract screening and 349 based on full-text assessment. The reasons for excluding the latter were: study not addressing the topic of interest (*n* = 56); study not a systematic review (*n* = 125); not studied in one of the EMR countries (*n* = 75); findings were aggregated and hence countries of the study could not be established (*n* = 21); EMR populations/ethnicities not living in the EMR countries (*n* = 16); or less than 30% of the studies included in the review addressed EMR population, a self-imposed restriction (*n* = 58). This process yielded a total of 89 systematic reviews for analyses (Fig. 1) (Supplementary file 2). Most studies (*n* = 69, 77.5%) reported searching two peer-reviewed databases (with or without supplementing with grey literature), while 15 studies (16.9%) reported searching one peer-reviewed database (with or without supplementing with grey literature). Four studies (4.5%) searched only grey literature, and one study (1.1%) reported searching “scientific databases”, without specifying the number or type.

The findings of the AMSTAR quality assessment are presented in Table 1. The summative score was skewed towards low AMSTAR values (median 3.0, range 0–9, interquartile range 2–4). Most of the reviews were judged as being of low AMSTAR quality (83.2%, *n* = 74). Close to 15% (*n* = 14) were of medium-quality, only one paper was judged as being of high-quality (summative score = 9), and none fulfilled *all* AMSTAR checklist items. The most frequently addressed item was whether the ‘characteristics of the included studies were provided’ (item 6, 95.5%, *n* = 85), followed by the ‘use of appropriate methods to combine study findings’ (item 9, 52.8%, *n* = 47), and the least was whether ‘potential conflicts of interest’ were included (0%). The remaining items were covered in less than a third of the papers. In the remaining analyses, the one study of high-quality AMSTAR was combined with those in the medium-quality AMSTAR category.

**Table 1:** Quality assessment using AMSTAR guidelines

**AMSTAR quality grouping (range)**		Number	Percent
Low (0–4)		74	83.1
Medium (5–8)		14	15.7
High (9–11)		1	1.1
**Item analyses**		% yes	
1.	Was an “a priori” design provided?	13.5	
2.	Was there duplicate study selection and data extraction?	21.4	
3.	Was a comprehensive literature search performed?	20.2	
4.	Was the status of publication (i.e., grey literature) used as an inclusion criterion?	19.1	
5.	Was a list of studies (included and excluded) provided?	3.4	
6.	Were the characteristics of the included studies provided?	95.5	
7.	Was the scientific quality of the included studies assessed and documented?	31.5	
8.	Was the scientific quality of the included studies used appropriately in formulating conclusions?	21.4	
9.	Were the methods used to combine the findings of studies appropriate?	52.8	
10.	Was the likelihood of publication bias assessed?	15.7	
11.	Were potential conflicts of interest included?	0.0	

**Fig. 2:**
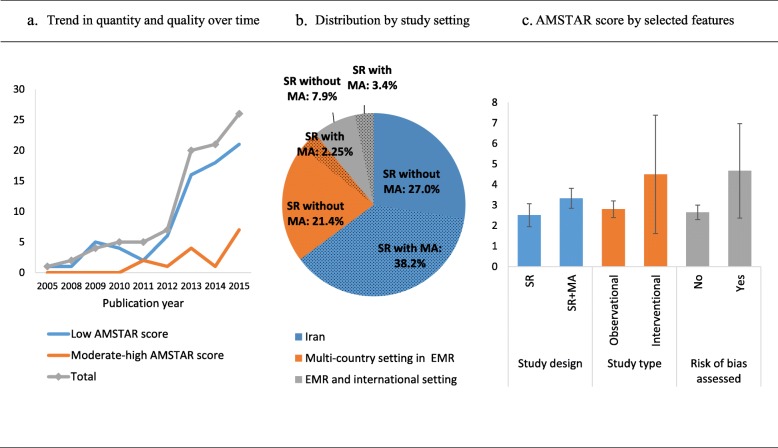
Trends in quantity and quality over time (**a**), distribution by study setting (**b**) and AMSTAR score by selected features (**c**)

The first NCD systematic review appearing in the EMR literature was in 2005. Annual number of publications increased over the years of study, with a significant surge in the latter 3 years between 2012 and 2015, during which close to three quarters of total reviews were published (Fig. 2a). Yet, the growth was mainly attributed to an increase in low-quality AMSTAR publications approaching in 2015 over four times the number of moderate/high-quality publications. Based on corresponding author affiliation, close to 72% (*n* = 64) of the reviews were carried out by an EMR-based author, mostly from Iran (56.2%, *n* = 50), followed by US (9.0%, *n* = 8) and UK-based (7.9%, *n* = 7) co-authors. A total of 39 studies (43.8%) included meta-analysis, and the remaining (56.2%, n = 50) were systematic reviews with no quantitative synthesis of the findings. Both types of reviews increased over time at equal pace. Close to 42% (*n* = 38) of the reviews were funded (35.9 and 7.1% from regional and international resources, respectively), 11.2% (*n* = 10) were not funded, and the remaining 46.1% (*n* = 41) did not report source of funding, if any (data not shown).

The distribution of the total number of studies included in each review was skewed towards higher values with a median of 21 and a range between 3 and 193 single studies. Most of the papers were reviews for studies conducted in a single country (64.0%, *n* = 57), the bulk of which is in Iran (53.9%, *n* = 48). Close to one fourth of the studies involved multi-country setting in the EMR region (24.7%, *n* = 22) and 11.2% (n = 10) involved additional settings from outside the EMR. Meta-analysis was more likely to be conducted when the review focused on studies on Iranian populations (Fig. 2b).

Most of the reviews focused on observational studies (78.7%), followed by intervention studies (12.4%). Critical appraisal of included studies was reported in 22.5% of the reviews. Reviews were more likely to be characterized by higher AMSTAR scores (±SD) when meta-analysis was performed compared when meta-analysis was not performed (3.4 ± 1.5 vs 2.6 ± 2.0; *p*-value = 0.034) and when the review included intervention studies compared to solely observational study designs (4.5 ± 3.1 vs 2.8 ± 1.6; p-value = 0.3596), and when critical appraisal was conducted (4.3 ± 2.3 vs 2.5 ± 1.5; *p*-value = 0.004) (Fig. 2c).

**Fig. 3:**
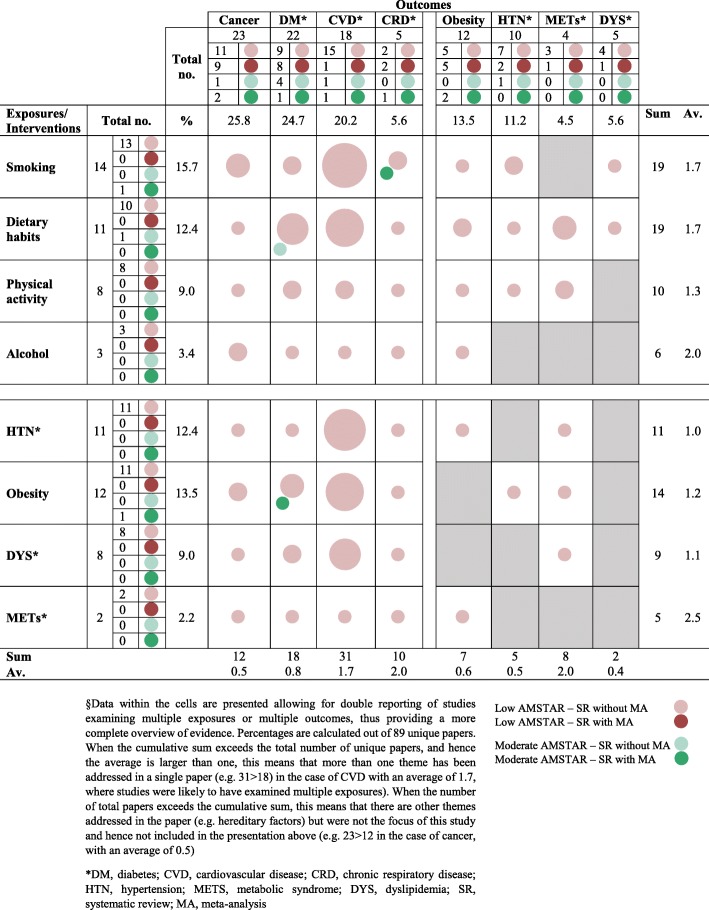
Gap Map showing frequency of publication by topic area of exposures and outcomes and by AMSTAR quality and study type (systematic review with/without meta-analysis)

Figure 3 presents the evidence gap map showing exposure(s) and outcome(s) appraised in the reviews, with a focus on the four key preventable behaviours, the four intermediary physiologic conditions, and the four main NCD outcomes. Cancer and diabetes were addressed in a large proportion of the reviews (*n* = 23, 25.8% and *n* = 22, 24.7%, respectively), with fewer tackling CVD (*n* = 18, 20.2%), and much less CRD (*n* = 5, 5.6%). Behavioral risk factors, including smoking, dietary habits, physical activity and alcohol, were examined in descending order in 15.7% (*n* = 14), 12.3% (*n* = 11), 9.0% (*n* = 8) and 3.4% (*n* = 3) of the reviews, respectively. These four risk factors and/or the four primary NCD conditions were addressed in 72% (*n*=64) of the total number of reports. Associations were mostly examined in relation to CVD (*n* = 31), followed by diabetes (n = 18). Physiologic factors and other intermediary variables in the casual pathway of NCD, including obesity, hypertension, dyslipidemia and METs, were featured as both exposures and outcomes in 43% (*n* = 38) of the reviews. It is worth noting that not all factors are clinically relevant to all outcomes, thus empty cells do not necessarily indicate clinically important gaps in the literature. Nevertheless, there remained significant areas where research is deficient, notably when it comes to the relation between behaviours and the intermediary variables in the causal pathway.

## Discussion

Guided by the ‘overview’ methodology and framework [18, 19], this study aimed to identify and assess the extent and quality of systematic reviews addressing NCDs and NCD risk factors in the EMR. The number of studies that fit our inclusion criteria totaled 89 publications, with the majority (64.0%) being conducted in one country, predominantly Iran. Whilst EMR countries have been increasingly productive in publishing NCD systematic reviews research over the past decade, yet this increase was characterized by a blatant lack of high-quality papers. The papers were overwhelmingly of low AMSTAR quality (83.2%), rarely incorporated an assessment of publication bias into the analysis (15.7%) or searched sources of the grey literature (19.1%), and only a handful provided a list of the included and excluded studies (3.4%). AMSTAR quality was significantly higher when meta-analysis was performed, and in reviews that included an assessment of publication bias. In addition, findings from the gap map analyses have shown a scarcity in the literature when it comes to associations between behavioral factors and intermediary physiologic factors leading to NCD conditions.

Worldwide, publication rates of systematic reviews have accelerated steadily in recent years, with one recent report counting a three-fold increase over the last decade [25]. Yet, this increase has been uneven across countries, with high-income countries producing about 200 times more reviews than low-income countries [26]. Similar observations were emphasised in a recent scoping review of NCD research in the region spanning the years 2000 until 2013, noting higher publication rates and the most rapid increases overtime in high-income countries [9]. With the exception of Iran, our findings of limited research productivity of systematic reviews on NCDs in the region (on average 2 publications per country) and of reviews evaluating interventions for the same NCD condition or in a single population align with the larger body of literature indicating a relative scarcity of high-evidence research [9, 17]. Reasons for the relatively lower quantity and quality of scientific productivity in the EMR include deficient research funding [27], instability of countries during conflicts [28, 29], poor research infrastructure, inadequate human resources and capacity, and difficulty in accessing biomedical databases and high-quality journals [30]. This is in addition to lack of international interest in studies targeting EMR challenges and concerns, and therefore lower chances of local manuscripts to be accepted in referred international journals [26].

Most NCDs are the result of four common preventable behaviours (tobacco use, physical inactivity, unhealthy diet, and the harmful use of alcohol) mediated by four metabolic/physiological changes (raised blood pressure, overweight/obesity, raised blood glucose and raised cholesterol). In our study, risk behaviors were commonly examined with various NCD conditions; yet, they rarely featured (only 7 reviews) in relation with physiologic factors and metabolic conditions, regarded key intermediary variables along the causal pathway for NCDs. Furthermore, with close to 35.9% of proportional mortality rate in the region being attributed to CVD, 11.7% to cancers, 8% to diabetes, and 3.8% to CRDs [31], CVD appeared to be an understudied topic when compared to cancer and diabetes. This concurs with similar observations made by Sibai et al. (9) in an earlier gap analysis of primary NCD studies conducted in the region showing a mismatch between disease burden and NCD research output, with a relative surplus of reports on cancer and a relative deficit of reports on CVDs. Research question prioritisation is required to guide resource allocation to areas of highest priority in support of policy and action [32].

While systematic reviews and overviews are meant to satisfy the demand from policy makers and practitioners for comprehensive evidence, a significant ingredient in realising these objectives include the availability of well-conducted and well-reported systematic reviews. Our assessment of systematic reviews from the region, using AMSTAR tool, shows an overall poor-quality output, raising serious concerns regarding the usability and usefulness of the research produced. A recent overview assessing socioeconomic inequalities in NCDs and their risk factors identified a total of 11 out of 22 systematic reviews with shortcomings in study selection and quality assessment process, with only one study being judged of high quality [33]. In their seminal paper, Chalmers and Glasziou [34] note that 85% of all health research is being avoidably “wasted”, and this is mostly attributed to inadequate description of the research questions, and poor or poorly explained methods and analysis [35]. Frameworks are increasingly being suggested to support quality enhancement initiatives at all study stages, from conceptualization of the research question to reporting of study results and knowledge translation [36, 37]. The limited number of well-conducted systematic reviews in our study highlights the need for more training in systematic review methods and calls upon reviewers and journal editors for more scrutiny of submitted manuscripts.

AMSTAR has been noted to be a reliable and valid measurement to assess the methodological quality of systematic reviews [38], and when compared to the Revised AMSTAR (R-AMSTAR), it has proven to be more trustworthy [39]. McKenzie and Brennan [40] in their recent editorial encourage submissions on the development and evaluation of conduct and methods for overview of systematic reviews. In our discussion, we report some concerns when using AMSTAR in the quality assessment of reviews. Three of its items (items 2, 5 and 7 in Table 1) are comprised of double-barrelled criteria, yet do not allow for more than one answer to be reported. For example in item 2, if a review reports duplicate study selection but not duplicate data extraction, it would not receive a favorable score. We appreciate the importance of methodological rigor in systematic reviews, however half scores need to be allowed to differentiate between the two steps and provide authors with more enlightened evaluation of the knowledge produced. Similarly, none of the reports scored positively on AMSTAR item 11 that required ‘conflict of interest’ for both the systematic review and the included primary studies to be addressed. This means that authors of systematic reviews who report on their own conflict of interest will not be credited if they do not report on the conflict of interest of the included studies.

Overall, we believe that some flexibility could be given while scoring AMSTAR and a consensus meeting for re-consideration of the items to be covered, with detailed assessment and analysis of AMSTAR notions being warranted. Nevertheless, these appraisals do not defend the overall low quality in systematic reviews from our region, which remains a major barrier to greater integration of evidence-based methodologies into practice. While our findings of significantly better-quality reviews in the case when meta-analysis was performed and when the review included an assessment of publication bias seem intuitive, further studies looking into the web of factors that may impact on the quality of reports are needed.

Our search strategy was designed to include as many relevant systematic reviews as possible, but we restricted eligibility criteria to systematic reviews published in English or French and we did not search local databases, so there might be other reviews in local or regional journals that we inadvertently omitted, particularly those written in Arabic or Farsi. However, regional scientific journals that publish in Arabic, specially on NCDs, are scarce and output from local journals are less likely to represent robust methodologies, and hence, our assessments provide a conservative estimate of the quality of systematic reviews in the region. On the other hand, our choice of a broad definition of systematic reviews counting-in studies reporting the use of one database for their search, while being more inclusive, may have skewed our results to including a high proportion of poor-quality reviews. Yet, to our knowledge, this is the first overview of studies addressing NCDs and NCD risk factors in the EMR; it reflects on the importance of producing high-quality systematic reviews and bridging research gaps.

## Conclusions

Our study identified several caveats for NCD systematic reviews in the EMR region. The lack of reviews from many countries in the region, together with the relatively low AMSTAR quality, and the deficient evidence in certain NCD topic areas, warrant the need for an informed research agenda and funding priorities, as well as capacity building and research infrastructure building for undertaking high-quality practice-evidence research in the region.

## Supplementary information

**Additional file 1.** Supplementary file 1. Search strategies and MESH terms.

**Additional file 2.** Supplementary file 2. Included studies.

## Data Availability

The original dataset used and analyzed during the current study are available from the corresponding author on reasonable request. We are also providing selected data on all the records in the supplementary file 2.

## Abbreviations

AMSTAR: Measurement tool to assess systematic reviews
CRD: Chronic respiratory diseases
CVD: Cardiovascular diseases
DM: Diabetes mellitus
EMR: Eastern Mediterranean Region
LMIC: Low- and middle-income countries
METs: Metabolic syndrome
NCD: Non-communicable diseases
WHO: World Health Organization
