# Supplementary material for: Non-communicable diseases research output in the Eastern Mediterranean region: an overview of systematic reviews
Source: BMC Med Res Methodol. 2020 Mar 20;20:68. doi: 10.1186/s12874-020-00924-0 (PMC7082905; doi:10.1186/s12874-020-00924-0)
Supplement: Supplementary file 1 — Additional file 1. Supplementary file 1. Search strategies and MESH terms. [file 12874_2020_924_MOESM1_ESM.docx]

**Supplementary file 1**

**Search strategies and MESH terms**

Database: Ovid MEDLINE(R) In-Process & Other Non-Indexed Citations and Ovid MEDLINE(R) <1946 to Present>

Search Strategy:

--------------------------------------------------------------------------------

1 exp glucose metabolism disorders/ or exp diabetes mellitus/ or exp prediabetic state/ or exp glycosuria/ or exp lipid metabolism disorders/ or exp dyslipidemias/ or exp hyperlipidemias/ or exp hypercholesterolemia/ or exp hyperlipidemia, familial combined/ or exp hyperlipoproteinemias/ or exp hypertriglyceridemia/ or exp hypolipoproteinemias/ or exp lipidoses/ or exp metabolic syndrome x/ (526543)

2 (Diabet* or ketoacidosis or (Donohue adj syndrom*) or glycosuria* or hyper?glyc?emi* or hba1c or prediabet* or (glucose adj2 (intoleran* or fasting* or disorder*))).tw. (530489)

3 ((metabolic adj2 (syndrome* or disturbance*)) or (insulin adj2 resistan*) or (deadly adj quartet) or (fasting adj2 (glycaemi* or sugar or glucose))).tw. (120556)

4 exp body weight changes/ or exp weight gain/ or exp weight loss/ or exp overweight/ or exp obesity/ or exp thinness/ or exp hypertriglyceridemic waist/ (218857)

5 (((lipid* or triglyceride* or cholesterol or lipoprotein*) adj2 (high or elevate* or increase*)) or hdl or ldl or vldl or (lipid* adj2 profile) or (lipid adj2 (metaboli* or disorder*)) or adiposit* or (abdominal adj2 fat) or hyper?lipid* or hypercholesterol* or dyslipid* or hypertriglyceride* or hyperlipoprote*).tw. (247464)

6 (Obesity or obese or Bmi or (body adj mass adj2 (index or indices)) or overweigh* or (percentage adj2 body adj2 fat) or (Waist adj hip adj ratio*) or (Waist adj2 circumference*) or (Weight adj2 (gain or change* or loss)) or (waist adj phenotype)).tw. (431859)

7 exp fasting/ or exp food habits/ or exp diet, diabetic/ or exp diet, carbohydrate-restricted/ or exp diet, cariogenic/ or exp diet, fat-restricted/ or exp diet, high-fat/ or exp diet, protein-restricted/ or exp diet, reducing/ or exp diet, sodium-restricted/ or exp diet, vegetarian/ or exp diet, western/ or exp energy intake/ (127793)

8 (((food or eating or dietary or diet*) adj2 (habit* or behavior?r* or Mediterranean)) or ((intake or reduc*) adj2 (salt or sodium))).tw. (32095)

9 exp cardiomyopathy, alcoholic/ or exp alcoholic intoxication/ or exp alcoholism/ or exp binge drinking/ or exp drinking behavior/ or exp alcohol abstinence/ or exp alcohol drinking/ or exp "tobacco use"/ or exp smoking/ or exp "tobacco use cessation"/ or exp smoking cessation/ or exp motor activity/ or exercise/ or exp physical fitness/ (503936)

10 ((Best adj2 buys) or ((physical or motor) adj2 (activit* or in?activit* or exercise*)) or exercise or exercising or (physical adj2 (fit or fitness*)) or walk or tobacco or smoke* or smoking or argile or water?pipe* or nargile or shisha or hookah or alcohol* or beverage* or (drink* adj2 (binge or heavy or intoxic* or driv*))).tw. (806818)

11 exp hypertension, pulmonary/ or exp asthma/ or exp bronchitis, chronic/ or exp pulmonary disease, chronic obstructive/ or exp pulmonary emphysema/ (185855)

12 ((chronic adj respiratory) or CRD or COPD or (chronic adj obstructive adj pulmonary) or emphysema* or (occupational adj lung) or asthma* or (respiratory adj2 (allerg* or hypersensitivit*))).tw. (201751)

13 exp cardiovascular diseases/ or exp cardiovascular abnormalities/ or exp cardiovascular infections/ or exp heart diseases/ or exp pregnancy complications, cardiovascular/ or exp vascular diseases/ (2117749)

14 (Cardiomegal* or cardiomyopath* or ((heart or cardia* or coronar* or myocardi* or vascular or arteriovenous*) adj3 (disease* or disorder* or malformation* or arrest or attack* or syndrome* or failure*)) or arrhythmia* or tachycardia* or endocard* or (infarction adj2 (myocardia* or cardiac or heart)) or isch?emi* or angina or shock or aneurysm or angiopath* or embolism or thrombosis or hypertensi* or phlebitis or atherosclerosis or arteritis).tw. (1603323)

15 exp Neoplasms/ (2919560)

16 (Neoplasm* or cyst* or tumo?r* or cancer* or carcinoma* or carcinoid or malignan* or metastat* or melanoma* or sarcoma* or leuk?emia* or leuc?emia* or lymphoma* or angiosarcoma* or astrocytoma* or glioma* or Cholangiocarcinoma* or Chondrosarcoma* or Craniopharyngioma* or Ependymoma* or Fibrosarcoma* or hemangioendothelioma* or Leiomyosarcoma* or Liposarcoma* or Medulloblastoma* or Meningioma* or Mesothelioma* or Neuroblastoma* or Oligodendroglioma* or Osteosarcoma* or Pheochromocytoma* or Pineoblastoma* or Rhabdomyosarcoma* or Thymoma* or adenocarcinoma*).tw. (3235663)

17 or/1-16 (7897394)

18 Middle East/ or exp africa, northern/ or exp egypt/ or exp libya/ or exp morocco/ or exp tunisia/ or exp djibouti/ or exp somalia/ or exp south sudan/ or exp sudan/ or exp afghanistan/ or exp bahrain/ or exp iran/ or exp iraq/ or exp jordan/ or exp kuwait/ or exp lebanon/ or exp oman/ or exp qatar/ or exp saudi arabia/ or exp syria/ or exp united arab emirates/ or exp yemen/ or exp pakistan/ (99848)

19 (Egypt* or libya* or morocc* or Tunisia* or Djibouti* or Somalia* or sudan* or Afghanistan* or Bahrain* or iran* or Iraq* or Jordan* or Kuwait* or lebanon or lebanese or oman* or Qatar* or Saudi* or Syria* or emirat* or yemen* or Pakistan*).tw. (131759)

20 ((north* adj2 Africa*) or MENA or levant or ((near or middle) adj east*) or gulf or (arab or arabian or arabs) or (palestin* or Gaza* or (west adj bank))).tw. (34522)

21 or/18-20 (181928)

22 search:.tw. (319032)

23 meta analysis.mp,pt. (118179)

24 review.pt. (2199221)

25 or/22-24 (2437930)

26 17 and 21 and 25 (2758)

27 limit 26 to yr="1996 - 2015" (2230)

Search Name: Overview of reviews may 2016

Last Saved: 06/5/2016 13:58:29.237

Description:

ID Search

#1 MeSH descriptor: [Metabolic Syndrome X] explode all trees

#2 MeSH descriptor: [Hyperlipidemias] explode all trees

#3 MeSH descriptor: [Body Weight Changes] explode all trees

#4 MeSH descriptor: [Diet] explode all trees

#5 MeSH descriptor: [Alcohol Drinking] explode all trees

#6 MeSH descriptor: [Smoking] explode all trees

#7 MeSH descriptor: [Tobacco Use Cessation] explode all trees

#8 MeSH descriptor: [Exercise] explode all trees

#9 insulin near/1 resistance:ti,ab,kw (Word variations have been searched)

#10 fasting near/1 glucose:ti,ab,kw (Word variations have been searched)

#11 Metabolic near/1 syndrome:ti,ab,kw (Word variations have been searched)

#12 Obesity or obese or Bmi or overweight or Weight:ti,ab,kw (Word variations have been searched)

#13 body near/1 mass:ti,ab,kw (Word variations have been searched)

#14 percentage near/1 body near/1 fat:ti,ab,kw (Word variations have been searched)

#15 Waist near/1 circumference:ti,ab,kw (Word variations have been searched)

#16 Waist near/1 hip near/1 ratio:ti,ab,kw (Word variations have been searched)

#17 lipid or triglyceride or cholesterol or lipoprotein or hdl or ldl or vldl adiposity or fat or hypercholesterol or dyslipidemia or hypertriglyceride or hyperlipoprotein:ti,ab,kw (Word variations have been searched)

#18 lipid near/1 profile:ti,ab,kw (Word variations have been searched)

#19 lipid near/1 metabolic near/1 disorder:ti,ab,kw (Word variations have been searched)

#20 food or eating or diet or salt or sodium or exercise or fitness or tobacco or smoke or argile or waterpipe or nargile or shisha or hookah or alcohol or beverage or drink:ti,ab,kw (Word variations have been searched)

#21 Best near/1 buys:ti,ab,kw (Word variations have been searched)

#22 physical near/1 activity:ti,ab,kw (Word variations have been searched)

#23 MeSH descriptor: [Lung Diseases, Obstructive] explode all trees

#24 MeSH descriptor: [Bronchitis, Chronic] explode all trees

#25 MeSH descriptor: [Pulmonary Emphysema] explode all trees

#26 MeSH descriptor: [Asthma] explode all trees

#27 CRD or COPD or emphysema or asthma:ti,ab,kw (Word variations have been searched)

#28 chronic near/1 respiratory:ti,ab,kw (Word variations have been searched)

#29 chronic near/1 obstructive near/1 pulmonary:ti,ab,kw (Word variations have been searched)

#30 occupational near/2 lung:ti,ab,kw (Word variations have been searched)

#31 pulmonary near/1 hypertension:ti,ab,kw (Word variations have been searched)

#32 obstructive near/1 lung:ti,ab,kw (Word variations have been searched)

#33 chronic near/1 bronchitis:ti,ab,kw (Word variations have been searched)

#34 MeSH descriptor: [Cardiovascular Diseases] explode all trees

#35 Cardiomegaly or cardiomyopathy or arrhythmia or tachycardia or phlebitis or arteritis or endocarditis or shock or aneurysm or angiopathy or embolism or thrombosis or hypertension or atherosclerosis or arrest or ischemia:ti,ab,kw (Word variations have been searched)

#36 heart near/1 disease:ti,ab,kw (Word variations have been searched)

#37 angina near/1 pectoris:ti,ab,kw (Word variations have been searched)

#38 Myocardial near/1 infarction:ti,ab,kw (Word variations have been searched)

#39 Blood near/1 pressure:ti,ab,kw (Word variations have been searched)

#40 vascular near/1 malformation:ti,ab,kw (Word variations have been searched)

#41 arteriovenous near/1 malformation:ti,ab,kw (Word variations have been searched)

#42 coronary near/1 disease:ti,ab,kw (Word variations have been searched)

#43 MeSH descriptor: [Glucose Metabolism Disorders] explode all trees

#44 Diabetes or glucose or ketone or ketoacidosis or gylcosuria or hyperglycemia or hyperinsulinim or hypoglycemia:ti,ab,kw (Word variations have been searched)

#45 glucose near/1 intolerance:ti,ab,kw (Word variations have been searched)

#46 MeSH descriptor: [Neoplasms] explode all trees

#47 Neoplasm or cyst or tumor or cancer carcinoma or carcinoid or malignancy or metastatis or melanoma or sarcoma or leukemia or lymphoma or angiosarcoma or astrocytoma or glioma or Cholangiocarcinoma or Chondrosarcoma or Craniopharyngioma or Ependymoma or Fibrosarcoma or hemangioendothelioma or Leiomyosarcoma or Liposarcoma or Medulloblastoma or Meningioma or Mesothelioma or Neuroblastoma or Oligodendroglioma or Osteosarcoma or Pheochromocytoma or Pineoblastoma or Rhabdomyosarcoma or Thymoma or adenocarcinoma:ti,ab,kw (Word variations have been searched)

#48 #1 or #2 or #3 or #4 or #5 or #6 or #7 or #8 or #9 or #10 or #11 or #12 or #13 or #14 or #15 or #16 or #17 or #18 or #19 or #20 or #21 or #22 or #23 or #24 or #25 or #26 or #27 or #28 or #29 or #30 or #31 or #32 or #33 or #34 or #35 or #36 or #37 or #38 or #39 or #40 or #41 or #42 or #43 or #44 or #45 or #46 or #47

#49 MeSH descriptor: [Afghanistan] explode all trees

#50 MeSH descriptor: [Bahrain] explode all trees

#51 MeSH descriptor: [Iran] explode all trees

#52 MeSH descriptor: [Iraq] explode all trees

#53 MeSH descriptor: [Jordan] explode all trees

#54 MeSH descriptor: [Kuwait] explode all trees

#55 MeSH descriptor: [Lebanon] explode all trees

#56 MeSH descriptor: [Oman] explode all trees

#57 MeSH descriptor: [Qatar] explode all trees

#58 MeSH descriptor: [Saudi Arabia] explode all trees

#59 MeSH descriptor: [Syria] explode all trees

#60 MeSH descriptor: [United Arab Emirates] explode all trees

#61 MeSH descriptor: [Yemen] explode all trees

#62 MeSH descriptor: [Pakistan] explode all trees

#63 MeSH descriptor: [Sudan] explode all trees

#64 MeSH descriptor: [Somalia] explode all trees

#65 MeSH descriptor: [Djibouti] explode all trees

#66 north near/1 Africa:ti,ab,kw (Word variations have been searched)

#67 middle near/1 east:ti,ab,kw (Word variations have been searched)

#68 west near/1 bank:ti,ab,kw (Word variations have been searched)

#69 MeSH descriptor: [Egypt] explode all trees

#70 MeSH descriptor: [Libya] explode all trees

#71 MeSH descriptor: [Morocco] explode all trees

#72 MeSH descriptor: [Tunisia] explode all trees

#73 Egypt or libya or morocc or Tunisia or Djibouti or Somalia or sudan or Afghanistan or Bahrain or iran or Iraq or Jordan or Kuwait or lebanon or lebanese or oman or Qatar or Saudi or Syria or emirat or yemen or Pakistan or palestine or Gaza or arab or mena or gulf:ti,ab,kw (Word variations have been searched)

#74 #49 or #50 or #51 or #52 or #53 or #54 or #55 or #56 or #57 or #58 or #59 or #60 or #61 or #62 or #63 or #64 or #65 or #66 or #67 or #68 or #69 or #70 or #71 or #72 or #73

#75 #48 and #74

Database: Epistemonikos May,2016

((Neoplasm* OR cyst* OR tumor* OR cancer* OR carcinoma* OR carcinoid OR malignan* OR metastat* OR "chronic pulmonary disease" OR "Chronic bronchitis" OR "chronic obstructive pulmonary disease" OR "pulmonary emphysema" OR "chronic respiratory disease" OR CRD OR COPD OR "occupational lung disease" OR "pulmonary hypertension" OR asthma* OR "respiratory allerg*" OR "respiratory hypersensitivit*" OR "Diabetes Mellitus" OR "Diabetes Type 1" OR "glucose intoleran*" OR "insulin resistnace" OR glucose* OR ketone* OR ketoacidosis OR gylcosuria OR hyperglycemi* Or glycemi* OR hyperinsulinim* OR hypoglycem* OR "glucose metabolis disorders" OR "prediabetic state" OR "lipid metabolism disorders" OR "dyslipidimia" OR "hyperlipidemia" OR "hypercholesterolemia" OR "familial combined hyperlipidemia" OR "hyperlipoproteinemias" OR "hypertriglyceridemia" OR "hypolipoproteinemias" OR lipidoses" OR "metabolic syndrome X" OR lipid* OR triglyceride* OR cholesterol OR lipoprotein* OR hdl OR ldl OR vldl OR fat OR "lipid profile" OR "lipid metabolic disorder" OR "body weight changes" OR "weight gain" OR "weight loss" OR overweight OR obesity OR thinness OR obese OR Bmi OR "body mass index" OR "percentage body fat" OR "Waist hip ratio" OR "Waist circumference" OR "waist phenotype" OR salt OR alcohol* OR tobacco OR smoking OR smoke OR "motor activity" OR exercise OR "physical fitness" OR "physical fitness" OR argile OR "water pipe” OR nargile OR shisha OR hookah OR "cardiovascular disease" OR "cardiovascular abnormalities" OR "cardiovascular infections" OR "heart diseases" OR "vascular disease" OR "vascular malformation" OR "arteriovenous malformation" OR "coronary disease" OR "blood pressure" OR "myocardial infarction" OR "angina pectoris" OR Cardiomegal* OR cardiomyopath* OR arrhythmia* OR tachycardia* OR endocard* OR myocard* OR arrest* OR diastolic OR systolic OR ischemi* OR shock OR aneurysm OR atherosclerosis OR angiopath* OR embolism OR thrombosis OR hypertension OR phlebitis OR arteritis) AND (Egypt* OR libya* OR morocco* OR tunisia* OR Djibouti* OR Somalia* OR sudan* OR Afghanistan* OR Bahrain* OR iran* OR Iraq* OR Jordan* OR Kuwait* OR Lebanon OR lebanese OR oman* OR Qatar* OR Syria* OR Saudi* OR yemen* OR Pakistan* OR Palestin* OR gaza OR Levant OR arab OR arabs OR arabian OR "united arab emarites" OR UAE OR "Saudi Arabia" OR "middle east" OR "Near east" OR gulf OR "northern Africa" OR "mena" OR "west bank"
