# Supplementary material for: Non-communicable diseases research output in the Eastern Mediterranean region: an overview of systematic reviews
Source: BMC Med Res Methodol. 2020 Mar 20;20:68. doi: 10.1186/s12874-020-00924-0 (PMC7082905; doi:10.1186/s12874-020-00924-0)
Supplement: Supplementary file 2 — Additional file 2. Supplementary file 2. Included studies. [file 12874_2020_924_MOESM2_ESM.docx]

**Supplementary II: Included studies**

| Author(s) | Year | Study design | Funding | Number of included studies | Setting | Population | Outcome(s) | Risk Factor(s) | Type of included studies |
| --- | --- | --- | --- | --- | --- | --- | --- | --- | --- |
| Abdi et al.[[1](#_ENREF_1)] | 2015 | SR | Funding not reported | 20 | Iran | General population | Other | NA | Interventional |
| Akbari et al.[[2](#_ENREF_2)] | 2014 | SR | Not funded | 17 | Iran | Adolescents and young adults | Other | NA | Observational |
| Akl et al.[[3](#_ENREF_3)] | 2011 | SR | Not funded | 38 | International and MENA | General and specific populations (high school students, university students, and pregnant women) | Hypertension | NA | Observational |
| Akl et al.[[4](#_ENREF_4)] | 2013 | SR | Funding not reported | 58 | International and MENA | General population | Other | NA | Mixed qualitative and quantitative |
| Al-Khudairy et al.[[5](#_ENREF_5)] | 2013 | SR | Funding not reported | 17 | Multiple MENA countries | Adults | Diabetes | Diet | Mixed |
| Alharbi et al.[[6](#_ENREF_6)] | 2014 | MA | Funded by King Saud University, Saudi Arabia | 36 | Multiple MENA countries | Adults | Diabetes; Obesity | NA | Not reported |
| Alhurishi et al.[[7](#_ENREF_7)] | 2011 | SR | Funded by King Saud University, Saudi Arabia | 10 | Multiple MENA countries | Women | Cancer | NA | Observational |
| Alhyas et al.[[8](#_ENREF_8)] | 2012 | SR | Funded by the UAE Ministry of research and higher education | 27 | Multiple MENA countries | General population | Diabetes | NA | Observational |
| Aljefree et al.[[9](#_ENREF_9)] | 2015 | SR | Funded by King Abdul Aziz University for Nutrition and Dietetics | 15 | Multiple MENA countries | Adults | Diabetes; CVD; Metabolic syndrome; Hypertension; Obesity; Dyslipidemia | Diet | Mixed |
| Amini et al.[[10](#_ENREF_10)] | 2009 | SR | Funded by Novo Nordisk Pars, Iran | 26 | Iran | Patients | Diabetes; CVD; Hypertension | NA | Observational |
| Amirkalali et al.[[11](#_ENREF_11)] | 2015 | MA | Funded by the Endocrinology and Metabolism Population Sciences Institute at the Tehran University of Medical Sciences | 28 | Iran | Adults | Metabolic syndrome | NA | Observational |
| Ardeshiri et al.[[12](#_ENREF_12)] | 2013 | MA | Funding not reported | 15 | Iran | Adults (15-64 yrs) | Other | NA | Observational |
| Ashtari et al.[[13](#_ENREF_13)] | 2015 | SR | Not funded | 21 | International and MENA | General population | Cancer | Obesity; Alcohol | Observational |
| Bamimore et al.[[14](#_ENREF_14)] | 2015 | SR | Funded by Seraj Kaki Chair for the study of genetic polymorphisms in cardiovascular disease and diabetes, King Abdulaziz University, Jeddah, Saudi Arabia; CIHR; Canada Chair; Saint Joseph University; Lebanese National Council for Scientific Research grants | Not mentioned | Multiple MENA countries | General population | Dyslipidemia | NA | Observational |
| Barakat et al.[[15](#_ENREF_15)] | 2012 | SR | Funding not reported | 6 | Single MENA country | General population | CVD | Hypertension; Obesity; Dyslipidemia; Smoking | Observational |
| Behrouz et al.[[16](#_ENREF_16)] | 2016 | SR | Funding not reported | 30 | Multiple MENA countries | Patients | CVD | Hypertension; Diet; Smoking | Observational |
| Benamer et al.[[17](#_ENREF_17)] | 2009 | SR | Funding not reported | 31 | Multiple MENA countries | General population | CVD | Hypertension; Diet | Observational |
| Benrahma et al.[[18](#_ENREF_18)] | 2014 | MA | Funded by NEPAD/NABNet New Partnership for Africa’s Development, North Africa Biosciences Network, and Joint WHO/EMRO-COMSTECH RAB&GH grants | 7 | Multiple MENA countries | Patients and controls | Diabetes | NA | Observational |
| Berhouma et al.[[19](#_ENREF_19)] | 2012 | MA | Funding not reported | 13 | Single MENA country | Tunisians with specified polymorphisms | Diabetes | NA | Observational |
| Borhani Haghighi et al.[[20](#_ENREF_20)] | 2012 | SR | Funding not reported | 7 | Iran | Patients | CVD | NA | Observational |
| Bos et al.[[21](#_ENREF_21)] | 2013 | SR | Funding not reported | 12 | Multiple MENA countries | Patients | Diabetes | Obesity | Observational |
| Boutayeb et al.[[22](#_ENREF_22)] | 2013 | SR | Funding not reported | 26 | Multiple MENA countries | General population | Cancer; Diabetes; CVD; CRD | Metabolic syndrome; Hypertension; Obesity; Dyslipidemia; Diet; Physical activity; Alcohol; Smoking | Observational |
| Chen et al.[[23](#_ENREF_23)] | 2015 | MA | Funding not reported | 13 | International and MENA | Patients and controls | CVD | NA | Observational |
| Cherbal et al.[[24](#_ENREF_24)] | 2013 | SR | Funded by the Algerian National Research Program (CNEPRU) | 11 | Multiple MENA countries | Maghrebian population/ families | Cancer | NA | Genetic |
| Deghaidy et al.[[25](#_ENREF_25)] | 2005 | MA | Funding not reported | 29 | Single MENA country | Patients | Cancer | NA | Observational |
| Djalalinia et al.[[26](#_ENREF_26)] | 2014 | SR | Funded by the Ministry of Health and Medical Education of Islamic Republic of Iran and Setad-e-Ejraie Farmane Imam | 53 | Iran | Children | Obesity | NA | Mixed qualitative and quantitative |
| Djalalinia et al.[[27](#_ENREF_27)] | 2015 | MA | Funding not reported | 19 | Iran | General population | Obesity | NA | Observational |
| Donnelly et al.[[28](#_ENREF_28)] | 2015 | SR | Funded by the Qatar National Research Fund under its National Priority Research Program | 6 | Multiple MENA countries | Women | Cancer | NA | Interventional |
| Eng et al.[[29](#_ENREF_29)] | 2014 | MA | Funded by the Senior Visiting Scientist Award by the International Agency for Research on Cancer (IARC) | 80 | International and MENA | Indigenous Populations: women | Cancer | NA | Observational |
| Forat-Yazdi et al.[[30](#_ENREF_30)] | 2015 | MA | Funded by the Shahid Sadoughi University of Medical Sciences, Yazd, Iran | 13 | Iran | Patients | Cancer | NA | Observational |
| Haghdoost et al.[[31](#_ENREF_31)] | 2008 | MA | Funded by the Kerman University of Medical Sciences (KUMS) | 29 | Iran | General population | Hypertension | NA | Observational |
| Haghdoost et al.[[32](#_ENREF_32)] | 2009 | MA | Funded by the Kerman University of Medical Sciences (KUMS) | 20 | Iran | General population | Diabetes | NA | Observational |
| Haghdoost et al.[[33](#_ENREF_33)] | 2013 | MA | Funded by the Research and Technology Deputy of Kerman University of Medical Science | 22 | Iran | Students | Other | NA | Observational |
| Hajian-Tilaki [[34](#_ENREF_34)] | 2015 | SR | Funding not reported | 43 | Iran | Adults | Metabolic syndrome | Hypertension; Obesity; Dyslipidemia; Diet; Physical activity | Observational |
| Halimi et al.[[35](#_ENREF_35)] | 2013 | MA | Funding not reported | 13 | Iran | Women | Other | NA | Observational |
| Hasan et al.[[36](#_ENREF_36)] | 2014 | SR | Funding not reported | 21 | Single MENA country | General population | CVD; Hypertension | Obesity; Physical activity; Smoking | Observational |
| Hosseini et al.[[37](#_ENREF_37)] | 2010 | SR | Funding not reported | 13 | Iran | General population | Other | Hypertension; Dyslipidemia; Smoking | Observational |
| Hovsepian et al.[[38](#_ENREF_38)] | 2015 | SR | Funded by the Ministry of Health and Medical Education of Islamic Republic of Iran, Setad-e-EjraieFarmane Imam | 26 | Iran | Children and adolescents | Dyslipidemia | NA | Observational |
| Irshad et al.[[39](#_ENREF_39)] | 2015 | MA | Funded by the College of Medicine Research Center, Deanship of Scientific Research, King Saud University, Riyadh, Saudi Arabia | 5 | Single MENA country | Patients and controls | Cancer | NA | Observational |
| Jafari-Adli et al.[[40](#_ENREF_40)] | 2014 | SR | Funding not reported | 193 | Iran | Adults and children | Obesity | NA | Observational |
| Jalilian et al.[[41](#_ENREF_41)] | 2015 | MA | Funded by the Ilam University of Medical Sciences | 30 | Iran | Women | Obesity | NA | Observational |
| Jalilvand et al.[[42](#_ENREF_42)] | 2014 | SR | Funding not reported | 61 | Iran | Patients and controls | Cancer | NA | Observational |
| Jalilvand et al.[[43](#_ENREF_43)] | 2015 | MA | Funded by the Tehran University of medical sciences | 20 | Iran | Women | Cancer | NA | Observational |
| Javanbakh et al.[[44](#_ENREF_44)] | 2015 | MA | Funded by the Iran University of Medical sciences | 6 | Iran | General population | Diabetes | NA | Mixed |
| Jazayeri et al.[[45](#_ENREF_45)] | 2013 | SR | Funding not reported | 9 | Iran | General population | Cancer | NA | Observational |
| Kelishadi et al.[[46](#_ENREF_46)] | 2014 | MA | Funded by the Isfahan University of Medical Sciences | 107 | Iran | Children and adolescents | Obesity | NA | Observational |
| Keyhani et al.[[47](#_ENREF_47)] | 2012 | MA | Funding not reported | 22 | Iran | Patients | Cancer | NA | Observational |
| Khodaeian et al.[[48](#_ENREF_48)] | 2015 | SR | Funding not reported | 88 | Iran | General population | Diabetes | NA | Observational |
| Khorasanizadeh et al.[[49](#_ENREF_49)] | 2013 | SR | Funded by the Tehran University of Medical Sciences; Mrs. Sohrabi Charity Fund in the Cancer Institute of Iran | 14 | Iran | Women | Cancer | NA | Observational |
| Kiadaliri et al.[[50](#_ENREF_50)] | 2013 | SR | Not funded | 46 | Iran | Patients | Other | Hypertension; Obesity; Dyslipidemia; Physical activity; Smoking | Mixed |
| Koonrung-sesomboon et al.[[51](#_ENREF_51)] | 2015 | MA | Funding not reported | 63 | International and MENA | Patients | Cancer | NA | Genetic |
| Laraqui et al.[[52](#_ENREF_52)] | 2015 | SR | Funding not reported | 13 | Multiple MENA countries | Patients | Cancer | NA | Genetic |
| Lasram et al.[[53](#_ENREF_53)] | 2014 | MA | Funded by NEPAD/NABNet T2D NA (New Partnership for Africa’s Development, North Africa Biosciences Network, Type 2 diabetes, North Africa); joint WHO/EMRO-COMSTECH RAB&GH Grants; the Tunisian Ministry of Higher Education and Scientific Research; the Tunisian Ministry of Health. | 3 | Single MENA country | Patients | Diabetes | NA | Not reported |
| Lehman et al.[[54](#_ENREF_54)] | 2009 | MA | Funded by a US National Science Foundation Graduate Research Fellowship and a Sigma Xi Grant-in-Aid of Research | 39 | Single MENA country | Population-based samples (non-high risk) and hepatocellular carcinoma cases | Cancer | NA | Genetic |
| Mabry et al.[[55](#_ENREF_55)] | 2010 | SR | Not funded | 4 | Multiple MENA countries | General population | Metabolic syndrome | Diet; Physical activity | Observational |
| Maleki et al.[[56](#_ENREF_56)] | 2015 | SR | Funded by the Tabriz University of Medical Sciences | 25 | Iran | Patients | Cancer | Smoking | Observational |
| Maziak et al.[[57](#_ENREF_57)] | 2015 | SR | Funded by the US Public Health Service Grants, Initiative for Cardiovascular Health Research in the Developing Countries (IC-Health), India | 3 | International and MENA | Smokers | Other | NA | Interventional |
| Meshkin et al.[[58](#_ENREF_58)] | 2013 | SR | Funded by the Iranian National Institute of Health and Health Technology Assessment Department of the Iranian Ministry of Health and Medical Education | 6 | Iran | Patients | Cancer | NA | Interventional |
| Meysamie et al.[[59](#_ENREF_59)] | 2012 | SR | Funding not reported | 83 | Iran | General population | Cancer; Diabetes; CVD; CRD; Hypertension; Dyslipidemia | Smoking | Observational |
| Mirmiran et al.[[60](#_ENREF_60)] | 2010 | SR | Funding not reported | 48 | Multiple MENA countries | Children | Obesity | NA | Observational |
| Mirzazadeh et al.[[61](#_ENREF_61)] | 2013 | MA | Funding not reported | 32 | Iran | Adults | Obesity | NA | Observational |
| Mohammadbeigi et al.[[62](#_ENREF_62)] | 2011 | MA | Funding not reported | 11 | Iran | Children | CRD | NA | Observational |
| Moosazadeh [[63](#_ENREF_63)] | 2013 | MA | Not funded | 17 | Iran | Adults (15-64 yrs) | Other | NA | Observational |
| Moosazadeh et al.[[64](#_ENREF_64)] | 2013 | MA | Funded by the Deputy of Research and Technology of Kerman University of Medical Sciences | 18 | Iran | General population | Other | NA | Observational |
| Moosazadeh et al.[[65](#_ENREF_65)] | 2014 | MA | Funding not reported | 12 | Iran | General population | Other | NA | Observational |
| Motlagh et al.[[66](#_ENREF_66)] | 2009 | SR | Funding not reported | 51 | Multiple MENA countries | General population | CVD | Hypertension; Obesity; Smoking | Observational |
| Mustafa [[67](#_ENREF_67)] | 2014 | SR | Funding not reported | 28 | Multiple MENA countries | General population | CVD | NA | Observational |
| Nazarzadeh et al.[[68](#_ENREF_68)] | 2013 | MA | Funded by the Zanjan University of Medical Science, Zanjan, Iran; the Psychosocial Injuries Research Center, Ilam, Iran. | 27 | Iran | Adolescents | Other | NA | Observational |
| Panahi et al.[[69](#_ENREF_69)] | 2015 | MA | Funded by the Baqiyatallah University of Medical Sciences and the Tabriz University of Medical Sciences | 7 | Iran | Individuals exposed to mustard gas | Cancer; CRD | NA | Observational |
| Peykari et al.[[70](#_ENREF_70)] | 2015 | SR | Funding not reported | 15 | Iran | General population | Diabetes | NA | Observational |
| Raad et al.[[71](#_ENREF_71)] | 2011 | MA | Funding not reported | 6 | Multiple MENA countries | Smokers and non-smokers | CRD | Smoking | Observational |
| Rahmani et al.[[72](#_ENREF_72)] | 2015 | MA | Funding not reported | 144 | Iran | Adults | Obesity | NA | Observational |
| Raza et al.[[73](#_ENREF_73)] | 2013 | MA | Funded by the Higher Education Commission of Pakistan and the Free University, Amsterdam, The Netherlands | 90 | Single MENA country | Indigenous and Immigrant | Diabetes; CVD; Hypertension; Obesity; Dyslipidemia | NA | Observational |
| Ruby et al.[[74](#_ENREF_74)] | 2015 | SR | Funded by the Research for Health in Humanitarian Crises (R2HC) Programme | 8 | International and MENA | Civilians affected by humanitarian crises Palestinian refugees in Jordan | Diabetes; CVD; Hypertension | NA | Mixed |
| Sarayani et al.[[75](#_ENREF_75)] | 2013 | SR | Not funded | 14 | Iran | Patients | Diabetes; CVD; Hypertension | NA | Mixed |
| Sefri et al.[[76](#_ENREF_76)] | 2014 | MA | Funded by the Passteur Institute of Morocco and a grant from the project NEPAD/NABNet | 21 | Single MENA country | Patients | Diabetes | NA | Genetic |
| Shaghaghi et al.[[77](#_ENREF_77)] | 2014 | SR | Funded by the Tabriz University of Medical Sciences | 62 | Iran | General population | Other | Hypertension; Obesity; Dyslipidemia; Diet; Physical activity; Smoking | Observational |
| Shayesteh et al.[[78](#_ENREF_78)] | 2013 | SR | Funded by the Research institute for Infectious Disease of Digestive System (RIDD) in Ahvaz Jundishapour University of Medical Science (AJUMS) and the Digestive Disease Research Center (DDRC) in Tehran University of Medical Science (TUMS) | 13 | Iran | Patients | Other | Smoking | Observational |
| Shobeiri et al.[[79](#_ENREF_79)] | 2015 | MA | Not funded | 24 | Iran | Women | Diabetes | Obesity | Observational |
| Sobhani et al.[[80](#_ENREF_80)] | 2014 | MA | Funding not reported | 21 | Iran | Patients | Other | NA | Observational |
| Sohal et al.[[81](#_ENREF_81)] | 2015 | SR | Funded by the Michael Smith Foundation for Health Research Career Scientist Award and the Alberta Innovates-Health Solutions Health Scholar Award | 20 | International and MENA | Patients | Diabetes | Diet; Physical activity | Observational |
| Tabatabaei-Malazy et al.[[82](#_ENREF_82)] | 2014 | MA | Not funded | 30 | Iran | General population | Other | NA | Observational |
| Tailakh et al.[[83](#_ENREF_83)] | 2014 | SR | Funding not reported | 13 | Multiple MENA countries | General population | Hypertension | NA | Observational |
| Toselli et al.[[84](#_ENREF_84)] | 2014 | SR | Funded by EU FP7/2007-2013 (EU and North African Migrants: Health and Health Systems (EUNAM)) | 30 | International and MENA | Adults | Obesity | Metabolic syndrome; Hypertension; Obesity; Diet; Physical activity; Alcohol; Smoking | Mixed |
| Tran et al.[[85](#_ENREF_85)] | 2010 | SR | Funding not reported | 26 | Multiple MENA countries | General population | CVD | NA | Observational |
| Veisani et al.[[86](#_ENREF_86)] | 2016 | MA | Funding not reported | 26 | Iran | Patients | Cancer | NA | Observational |
| Yaghoubi et al.[[87](#_ENREF_87)] | 2012 | SR | Funding not reported | 18 | Iran | Patients | CVD | Hypertension; Dyslipidemia; Smoking | Observational |
| Yarhusseini et al.[[88](#_ENREF_88)] | 2014 | MA | Funding not reported | 8 | Iran | Patients | Cancer | NA | Not reported |
| Zabetian et al.[[89](#_ENREF_89)] | 2013 | SR | Not funded | 110 | Multiple MENA countries | General population | Diabetes; CVD | Obesity; Dyslipidemia; Diet | Observational |

1. Abdi J, Eftekhar H, Estebsari F, Sadeghi R. Theory-based interventions in physical activity: a systematic review of literature in Iran. Global journal of health science. 2015;7(3):215.

2. Akbari F, Azadbakht L. A systematic review on diet quality among Iranian youth: focusing on reports from Tehran and Isfahan. Archives of Iranian medicine. 2014;17(8):574-84.

3. Akl EA, Gunukula SK, Aleem S, Obeid R, Jaoude PA, Honeine R, et al. The prevalence of waterpipe tobacco smoking among the general and specific populations: a systematic review. BMC public health. 2011;11:244.

4. Akl EA, Jawad M, Lam WY, Co CN, Obeid R, Irani J. Motives, beliefs and attitudes towards waterpipe tobacco smoking: a systematic review. Harm reduction journal. 2013;10:12.

5. Al-Khudairy L, Stranges S, Kumar S, Al-Daghri N, Rees K. Dietary factors and type 2 diabetes in the Middle East: what is the evidence for an association?--a systematic review. Nutrients. 2013;5(10):3871-97.

6. Alharbi NS, Almutari R, Jones S, Al-Daghri N, Khunti K, de Lusignan S. Trends in the prevalence of type 2 diabetes mellitus and obesity in the Arabian Gulf States: systematic review and meta-analysis. Diabetes Res Clin Pract. 2014;106(2):e30-3.

7. Alhurishi S, Lim JN, Potrata B, West R. Factors influencing late presentation for breast cancer in the middle East: a systematic review. Asian Pac J Cancer Prev. 2011;12(6):1597-600.

8. Alhyas L, McKay A, Majeed A. Prevalence of type 2 diabetes in the States of the co-operation council for the Arab States of the Gulf: a systematic review. PloS one. 2012;7(8):e40948.

9. Aljefree N, Ahmed F. Association between dietary pattern and risk of cardiovascular disease among adults in the Middle East and North Africa region: a systematic review. Food & nutrition research. 2015;59:27486.

10. Amini M, Parvaresh E. Prevalence of macro- and microvascular complications among patients with type 2 diabetes in Iran: a systematic review. Diabetes Res Clin Pract. 2009;83(1):18-25.

11. Amirkalali B, Fakhrzadeh H, Sharifi F, Kelishadi R, Zamani F, Asayesh H, et al. Prevalence of Metabolic Syndrome and Its Components in the Iranian Adult Population: A Systematic Review and Meta-Analysis. Iranian Red Crescent medical journal. 2015;17(12):e24723.

12. Ardeshiri MJ, Moosazadeh M, Masouleh MF, Masouleh MF, Kiani A, Fakhri M. Prevalence of smoking in 15-64 years old population of north of Iran: meta-analysis of the results of non-communicable diseases risk factors surveillance system. Acta Medica Iranica. 2013;51(7):494-500.

13. Ashtari S, Pourhoseingholi MA, Sharifian A, Zali MR. Hepatocellular carcinoma in Asia: Prevention strategy and planning. World journal of hepatology. 2015;7(12):1708-17.

14. Bamimore MA, Zaid A, Banerjee Y, Al-Sarraf A, Abifadel M, Seidah NG, et al. Familial hypercholesterolemia mutations in the Middle Eastern and North African region: a need for a national registry. Journal of clinical lipidology. 2015;9(2):187-94.

15. Barakat H, Barakat H, Baaj MK. CVD and obesity in transitional Syria: a perspective from the Middle East. Vascular health and risk management. 2012;8:145-50.

16. Behrouz R, Powers CJ. Epidemiology of classical risk factors in stroke patients in the Middle East. European journal of neurology. 2016;23(2):262-9.

17. Benamer HT, Grosset D. Stroke in Arab countries: a systematic literature review. Journal of the neurological sciences. 2009;284(1-2):18-23.

18. Benrahma H, Charoute H, Lasram K, Boulouiz R, Atig RK, Fakiri M, et al. Association analysis of IGF2BP2, KCNJ11, and CDKAL1 polymorphisms with type 2 diabetes mellitus in a Moroccan population: a case-control study and meta-analysis. Biochemical genetics. 2014;52(9-10):430-42.

19. Berhouma R, Kouidhi S, Ammar M, Abid H, Baroudi T, Ennafaa H, et al. Genetic susceptibility to type 2 diabetes: a global meta-analysis studying the genetic differences in Tunisian populations. Human biology. 2012;84(4):423-35.

20. Borhani Haghighi A, Ashjazadeh N, Safari A, Cruz-Flores S. Cerebral venous sinus thrombosis in iran: cumulative data, shortcomings and future directions. Iranian Red Crescent medical journal. 2012;14(12):805-10.

21. Bos M, Agyemang C. Prevalence and complications of diabetes mellitus in Northern Africa, a systematic review. BMC public health. 2013;13:387.

22. Boutayeb A, Boutayeb S, Boutayeb W. Multi-morbidity of non communicable diseases and equity in WHO Eastern Mediterranean countries. International journal for equity in health. 2013;12(1):60.

23. Chen A, Li G, Liu Y. Association between copper levels and myocardial infarction: a meta-analysis. Inhalation toxicology. 2015;27(5):237-46.

24. Cherbal F, Bakour R, Adane S, Boualga K. BRCA1 and BRCA2 germline mutation spectrum in hereditary breast/ovarian cancer families from Maghrebian countries. Breast disease. 2013;34(1):1-8.

25. Deghaidy AA, Nofal LM, Abd-Elmoneium SE, Mahdy NH. Meta-analysis of survival models of lung cancer. The Journal of the Egyptian Public Health Association. 2005;80(1-2):77-126.

26. Djalalinia S, Kelishadi R, Qorbani M, Peykari N, Kasaeian A, Saeedi Moghaddam S, et al. Suggestions for better data presentation in papers: an experience from a comprehensive study on national and sub-national trends of overweight and obesity. Archives of Iranian medicine. 2014;17(12):830-6.

27. Djalalinia S, Peykari N, Qorbani M, Larijani B, Farzadfar F. Inequality of obesity and socioeconomic factors in Iran: a systematic review and meta- analyses. Medical journal of the Islamic Republic of Iran. 2015;29:241.

28. Donnelly TT, Hwang J. Breast cancer screening interventions for Arabic women: a literature review. Journal of immigrant and minority health. 2015;17(3):925-39.

29. Eng A, McCormack V, dos-Santos-Silva I. Receptor-defined subtypes of breast cancer in indigenous populations in Africa: a systematic review and meta-analysis. PLoS medicine. 2014;11(9):e1001720.

30. Forat-Yazdi M, Neamatzadeh H, Sheikhha MH, Zare-Shehneh M, Fattahi M. BRCA1 and BRCA2 common mutations in iranian breast cancer patients: a meta analysis. Asian Pac J Cancer Prev. 2015;16(3):1219-24.

31. Haghdoost AA, Sadeghirad B, Rezazadehkermani M. Epidemiology and heterogeneity of hypertension in Iran: a systematic review. Archives of Iranian medicine. 2008;11(4):444-52.

32. Haghdoost AA, Rezazadeh-Kermani M, Sadghirad B, Baradaran HR. Prevalence of type 2 diabetes in the Islamic Republic of Iran: systematic review and meta-analysis. Eastern Mediterranean health journal = La revue de sante de la Mediterranee orientale = al-Majallah al-sihhiyah li-sharq al-mutawassit. 2009;15(3):591-9.

33. Haghdoost AA, Moosazadeh M. The prevalence of cigarette smoking among students of Iran's universities: A systematic review and meta-analysis. Journal of research in medical sciences : the official journal of Isfahan University of Medical Sciences. 2013;18(8):717-25.

34. Hajian-Tilaki K. Metabolic syndrome and its associated risk factors in Iranian adults: A systematic review. Caspian journal of internal medicine. 2015;6(2):51-61.

35. Halimi L, Haghdoost AA, Mohammad Alizadeh S. Prevalence of cigarette smoking among Iranian women: a systematic review and meta-analysis. Medical journal of the Islamic Republic of Iran. 2013;27(3):132-40.

36. Hasan DM, Emeash AH, Mustafa SB, Abdelazim GE, El-din AA. Hypertension in Egypt: a systematic review. Current hypertension reviews. 2014;10(3):134-41.

37. Hosseini AA, Sobhani-Rad D, Ghandehari K, Benamer HT. Frequency and clinical patterns of stroke in Iran - Systematic and critical review. BMC neurology. 2010;10:72.

38. Hovsepian S, Kelishadi R, Djalalinia S, Farzadfar F, Naderimagham S, Qorbani M. Prevalence of dyslipidemia in Iranian children and adolescents: A systematic review. Journal of research in medical sciences : the official journal of Isfahan University of Medical Sciences. 2015;20(5):503-21.

39. Irshad M, Mandal RK, Al-Drees A, Khalil MS, Abdulghani HM. No Evidence of Association of the Arg72Pro p53 Gene Polymorphism with Cancer Risk in the Saudi Population: a Meta-Analysis. Asian Pac J Cancer Prev. 2015;16(14):5663-7.

40. Jafari-Adli S, Jouyandeh Z, Qorbani M, Soroush A, Larijani B, Hasani-Ranjbar S. Prevalence of obesity and overweight in adults and children in Iran; a systematic review. Journal of diabetes and metabolic disorders. 2014;13(1):121.

41. Jalilian A, Kiani F, Sayehmiri F, Sayehmiri K, Khodaee Z, Akbari M. Prevalence of polycystic ovary syndrome and its associated complications in Iranian women: A meta-analysis. Iranian journal of reproductive medicine. 2015;13(10):591.

42. Jalilvand S, Shoja Z, Hamkar R. Human papillomavirus burden in different cancers in Iran: a systematic assessment. Asian Pac J Cancer Prev. 2014;15(17):7029-35.

43. Jalilvand S, Shoja Z, Nourijelyani K, Tohidi HR, Hamkar R. Meta‐analysis of type‐specific human papillomavirus prevalence in Iranian women with normal cytology, precancerous cervical lesions and invasive cervical cancer: Implications for screening and vaccination. Journal of medical virology. 2015;87(2):287-95.

44. Javanbakht M, Mashayekhi A, Baradaran HR, Haghdoost A, Afshin A. Projection of diabetes population size and associated economic burden through 2030 in Iran: evidence from micro-simulation Markov model and Bayesian meta-analysis. PloS one. 2015;10(7):e0132505.

45. Jazayeri SB, Rahimi-Movaghar V, Shokraneh F, Saadat S, Ramezani R. Epidemiology of primary CNS tumors in Iran: a systematic. Asian Pacific Journal of Cancer Prevention. 2013;14(6):3979-85.

46. Kelishadi R, Haghdoost A-A, Sadeghirad B, Khajehkazemi R. Trend in the prevalence of obesity and overweight among Iranian children and adolescents: a systematic review and meta-analysis. Nutrition. 2014;30(4):393-400.

47. Keyhani E, Muhammadnejad A, Karimlou M. Prevalence of HER-2-positive invasive breast cancer: a systematic review from Iran. Asian Pacific Journal of Cancer Prevention. 2012;13(11):5477-82.

48. Khodaeian M, Enayati S, Tabatabaei-Malazy O, Amoli MM. Association between genetic variants and diabetes mellitus in Iranian populations: a systematic review of observational studies. Journal of diabetes research. 2015;2015.

49. Khorasanizadeh F, Hassanloo J, Khaksar N, Taheri SM, Marzaban M, Rashidi BH, et al. Epidemiology of cervical cancer and human papilloma virus infection among Iranian women—Analyses of national data and systematic review of the literature. Gynecologic oncology. 2013;128(2):277-81.

50. Kiadaliri AA, Najafi B, Mirmalek-Sani M. Quality of life in people with diabetes: a systematic review of studies in Iran. Journal of Diabetes & Metabolic Disorders. 2013;12(1):54.

51. Koonrungsesomboon N, Wadagni AC, Mbanefo EC. Molecular markers and Schistosoma-associated bladder carcinoma: A systematic review and meta-analysis. Cancer epidemiology. 2015;39(4):487-96.

52. Laraqui A, Uhrhammer N, El Rhaffouli H, Sekhsokh Y, Lahlou-Amine I, Bajjou T, et al. BRCA genetic screening in Middle Eastern and North African: mutational spectrum and founder BRCA1 mutation (c. 798_799delTT) in North African. Disease markers. 2015;2015.

53. Lasram K, Ben Halim N, Hsouna S, Kefi R, Arfa I, Ghazouani W, et al. Evidence for Association of the E23K Variant of KCNJ11 Gene with Type 2 Diabetes in Tunisian Population: Population-Based Study and Meta-Analysis. BioMed Research International. 2014;2014:265274.

54. Lehman EM, Wilson ML. Epidemiology of hepatitis viruses among hepatocellular carcinoma cases and healthy people in Egypt: A systematic review and meta‐analysis. International journal of Cancer. 2009;124(3):690-7.

55. Mabry R, Reeves M, Eakin E, Owen N. Gender differences in prevalence of the metabolic syndrome in Gulf Cooperation Council Countries: a systematic review. Diabetic Medicine. 2010;27(5):593-7.

56. Maleki D, Ghojazadeh M, Mahmoudi S-S, Mahmoudi S-M, Pournaghi-Azar F, Torab A, et al. Epidemiology of oral cancer in Iran: a systematic review. Asian Pacific journal of cancer prevention. 2015;16(13):5427-32.

57. Maziak W, Jawad M, Jawad S, Ward KD, Eissenberg T, Asfar T. Interventions for waterpipe smoking cessation. The Cochrane database of systematic reviews. 2015;7:CD005549.

58. Hashemi-Meshkini A, Keshavarz K, Gharibnaseri Z, Kheirandish M, Kebriaeezadeh A, Nikfar S, et al. Cost-effectiveness analysis review of exemestane in the treatment of primary and advanced breast cancer. Archives of medical science: AMS. 2013;9(3):472.

59. Meysamie A, Ghaletaki R, Zhand N, Abbasi M. Cigarette smoking in iran. Iran J Public Health. 2012;41(2):1-14.

60. Mirmiran P, Sherafat Kazemzadeh R, Jalali Farahani S, Azizi F. Childhood obesity in the Middle East: a review. 2010.

61. Mirzazadeh A, Salimzadeh H, Arabi M, Navadeh S, Hajarizadeh B, Haghdoost AA. Trends of Obesity in Iranian Adults from 1990s to late 2000s; a Systematic Review and Meta-analysis. Middle East journal of digestive diseases. 2013;5(3):151-7.

62. Mohammadbeigi A, Hassanzadeh J, Mousavizadeh A. Prevalence of asthma in elementary school age children in Iran--a systematic review and meta analysis study. 2011.

63. Moosazadeh M. Meta-Analysis of Prevalence of Smoking in 15-64-year-old Population of West of Iran. International journal of preventive medicine. 2013;4(10):1108-14.

64. Moosazadeh M, Ziaaddini H, Mirzazadeh A, Ashrafi-Asgarabad A, Haghdoost AA. Meta-analysis of smoking prevalence in Iran. Addiction & health. 2013;5(3-4):140.

65. Moosazadeh M, Salami F, Movahednia M, Amiri MM, Afshari M. Prevalence of smoking in northwest Iran: a meta-analysis. Electronic physician. 2014;6(1):734-40.

66. Motlagh B, O'donnell M, Yusuf S. Prevalence of cardiovascular risk factors in the Middle East: a systematic review. European Journal of Cardiovascular Prevention & Rehabilitation. 2009;16(3):268-80.

67. Mustafa KN. Takayasu's arteritis in Arabs. Clinical rheumatology. 2014;33(12):1777-83.

68. Nazarzadeh M, Bidel Z, Ayubi E, Bahrami A, Jafari F, Mohammadpoorasl A, et al. Smoking status in Iranian male adolescents: a cross-sectional study and a meta-analysis. Addictive behaviors. 2013;38(6):2214-8.

69. Panahi Y, Gholami N, Ghojazadeh M, Moslemi F, Naghavi-Behzad M, Azami-Aghdash S, et al. complications and carcinogenic effects of mustard gas-a systematic review and meta-analysis in Iran. Asian Pac J Cancer Prev. 2015;16:7567-73.

70. Peykari N, Djalalinia S, Qorbani M, Sobhani S, Farzadfar F, Larijani B. Socioeconomic inequalities and diabetes: A systematic review from Iran. Journal of diabetes and metabolic disorders. 2015;14:8.

71. Raad D, Gaddam S, Schunemann HJ, Irani J, Abou Jaoude P, Honeine R, et al. Effects of water-pipe smoking on lung function: a systematic review and meta-analysis. Chest. 2011;139(4):764-74.

72. Rahmani A, Sayehmiri K, Asadollahi K, Sarokhani D, Islami F, Sarokhani M. Investigation of the Prevalence of Obesity in Iran: a Systematic Review and Meta-Analysis Study. Acta Med Iran. 2015;53(10):596-607.

73. Raza Q, Doak CM, Khan A, Nicolaou M, Seidell JC. Obesity and cardiovascular disease risk factors among the indigenous and immigrant Pakistani population: a systematic review. Obesity facts. 2013;6(6):523-35.

74. Ruby A, Knight A, Perel P, Blanchet K, Roberts B. The Effectiveness of Interventions for Non-Communicable Diseases in Humanitarian Crises: A Systematic Review. PLoS One. 2015;10(9):e0138303.

75. Sarayani A, Jahangard-Rafsanjani Z, Hadjibabaie M, Ahmadvand A, Javadi M, Gholami K. A comprehensive review of adherence to diabetes and cardiovascular medications in Iran; implications for practice and research. Journal of diabetes and metabolic disorders. 2013;12(1):57.

76. Sefri H, Benrahma H, Charoute H, Rouba H, Lyoussi B, Nourlil J, et al. TNF A− 308G> A polymorphism in Moroccan patients with type 2 diabetes mellitus: a case–control study and meta-analysis. Molecular biology reports. 2014;41(9):5805-11.

77. Shaghaghi A, Ahmadi A. Evidence Gap on the Prevalence of Non-conventional Risk Factors for Type 2 Diabetes in Iran. Osong public health and research perspectives. 2014;5(5):292-7.

78. Shayesteh AA, Saberifirozi M, Abedian S, Sebghatolahi V. Epidemiological, Demographic, and Colonic Extension ofUlcerative Colitis in Iran: A Systematic Review. Middle East journal of digestive diseases. 2013;5(1):29-36.

79. Jafari-Shobeiri M, Ghojazadeh M, Azami-Aghdash S, Naghavi-Behzad M, Piri R, Pourali-Akbar Y, et al. Prevalence and risk factors of gestational diabetes in Iran: a systematic review and meta-analysis. Iranian journal of public health. 2015;44(8):1036.

80. Sobhani S, Asayesh H, Sharifi F, Djalalinia S, Baradaran HR, Arzaghi SM, et al. Prevalence of diabetic peripheral neuropathy in Iran: a systematic review and meta-analysis. Journal of Diabetes & Metabolic Disorders. 2014;13(1):97.

81. Sohal T, Sohal P, King-Shier KM, Khan NA. Barriers and facilitators for type-2 diabetes management in South Asians: a systematic review. PloS one. 2015;10(9):e0136202.

82. Tabatabaei-Malazy O, Qorbani M, Samavat T, Sharifi F, Larijani B, Fakhrzadeh H. Prevalence of dyslipidemia in Iran: a systematic review and meta-analysis study. International journal of preventive medicine. 2014;5(4):373.

83. Tailakh A, Evangelista LS, Mentes JC, Pike NA, Phillips LR, Morisky DE. Hypertension prevalence, awareness, and control in A rab countries: A systematic review. Nursing & health sciences. 2014;16(1):126-30.

84. Toselli S, Gualdi-Russo E, Boulos DN, Anwar WA, Lakhoua C, Jaouadi I, et al. Prevalence of overweight and obesity in adults from North Africa. European journal of public health. 2014;24 Suppl 1:31-9.

85. Tran J, Mirzaei M, Anderson L, Leeder SR. The epidemiology of stroke in the Middle East and North Africa. Journal of the neurological sciences. 2010;295(1-2):38-40.

86. Veisani Y, Delpisheh A. Survival rate of gastric cancer in Iran; a systematic review and meta-analysis. Gastroenterology and hepatology from bed to bench. 2016;9(2):78.

87. Yaghoubi A, Tabrizi J-S, Mirinazhad M-M, Azami S, Naghavi-Behzad M, Ghojazadeh M. Quality of life in cardiovascular patients in Iran and factors affecting it: A systematic review. Journal of cardiovascular and thoracic research. 2012;4(4):95.

88. Yarhusseini A, Sharifzadeh L, Delpisheh A, Veisani Y, Sayehmiri F, Sayehmiri K. Survival rate of esophageal carcinoma in Iran-a systematic review and meta-analysis. Iranian journal of cancer prevention. 2014;7(2):61.

89. Zabetian A, Keli HM, Echouffo-Tcheugui JB, Narayan KV, Ali MK. Diabetes in the middle east and north Africa. Diabetes research and clinical practice. 2013;101(2):106-22.
